# AI-simulated clinical consultations: Assessing the potential of ChatGPT to support medical training

**DOI:** 10.1136/archdischild-2025-329846

**Published:** 2026-03-06

**Authors:** Arpita Saggar, Vania Dimitrova, Duygu Sarikaya, David Hogg, Jonathan C Darling

**Affiliations:** 1 Computer Science, University of Leeds, Leeds, UK; 2 Division of Women's and Children’s Health, University of Leeds, Leeds, UK; 3 Paediatric Medicine, Leeds General Infirmary, Leeds, UK

**Keywords:** Paediatrics, Technology, Information Technology

## Abstract

**Background:**

Simulated medical scenarios are useful for evaluating and developing clinical competencies but scheduling them is expensive and time-consuming. Large language models show promise in role-playing tasks. We investigated the fidelity with which ChatGPT can mimic patients, clinicians and examiners in educational settings.

**Objective:**

To determine the realism with which ChatGPT can portray patient, doctor and examiner roles, and the utility of these agents in clinical education.

**Method:**

We selected four paediatric scenarios from mock objective structured clinical examinations (OSCEs) and set up separate patient, doctor and examiner ChatGPT agents for each. The patient and doctor agents conversed with each other in written format. The examiner agent marked the doctor agent based on this conversation. Patients and clinicians familiar with the OSCE assessed the dialogues.

**Results:**

The patient agent was judged to be true to character most of the time and good at expressing emotion. The doctor agent was reported to be an effective communicator but occasionally used jargon. Both agents tended to produce repetitive responses which undermined realism. The examiner agent had good correlation with human clinicians. There was moderate support for using the simulated interactions for educational purposes.

**Conclusion:**

Although the realism of the agents can be improved, ChatGPT can generate plausible proxies of participants in medical scenarios and could be useful for complementing standardised patient-based training.

**WHAT IS ALREADY KNOWN ON THIS TOPIC**

- Large language model-based agents show promise for portraying clinical roles and supporting simulation-based learning. Doctor agents provide correct diagnoses most of the time, while patient agents can accurately relay role information such as medical history or symptoms.

**WHAT THIS STUDY ADDS**

- There is scope for improvement in the realism and authenticity of the conversations produced by GPT patient and doctor agents. Notable issues included a tendency to produce repetitive and verbose responses, and an inability to accurately convey the hesitation shown by real patients.
- Disparities observed between (human) patient and clinician assessment for the GPT agents suggest that diverse viewpoints are needed to fully capture the experiential learning associated with clinical communication.

**HOW THIS STUDY MIGHT AFFECT RESEARCH, PRACTICE OR POLICY**

- Low fidelity of GPT simulations for difficult or challenging medical scenarios necessitates human oversight and correction for artificial intelligence (AI) deployed in educational settings.
- The impact of AI on medical education is likely to increase in the future, which necessitates promoting AI literacy among educators and students.

## Introduction

 The development of effective clinical communication skills is vital to ensure the delivery of quality healthcare. Simulated medical scenarios have been widely used in medical education settings for decades. They are a reliable method for teaching these skills and evaluating the competencies of doctors,^1^ through assessments such as the objective structured clinical examination (OSCE).^2^ In these simulations, patient roles are typically fulfilled by actors who have been coached to present like real patients, using predetermined scenarios.^3^ However, recruiting and training standardised patients (SPs) is time-consuming. Scheduling interactions also requires administrators to maintain records and book the SPs. Additionally, the total cost of remuneration may be substantial for large-scale training and assessment,^4 5^ which further reduces the feasibility of such arrangements. There is increasing pressure on clinical placement time due to workforce expansion.^6^

Recently, generative artificial intelligence (AI) has shown remarkable success in producing realistic text, speech and video. These models could be integrated to develop multimodal agents that can supplement traditional training, but this remains technically challenging. We note that text serves as the foundational modality for these agents, providing the basis for subsequent speech generation and facial animation. We therefore focus on language models as a starting point to assess how AI can support medical education. Large language models (LLMs) are tuned to follow instructions, which include adopting a given persona over the course of an interaction.^7 8^ They can portray specific roles, making them ideal for simulation-based teaching and assessment. However, the utility and reliability of these models in the medical classroom remain unclear.^9^ Many previous efforts focus on measuring the performance of LLMs on standardised medical examinations.^10^,^14^ While useful for establishing clinical knowledge, these evaluations rely solely on quantitative metrics and fail to explore the capabilities of LLMs in open-ended discourse. Other studies that leverage LLM-based doctor agents tend to prioritise performance on rigid attributes like information gathering ability, diagnostic reasoning or compliance to guidelines.^15^,^17^ However, effective clinical communication extends beyond providing accurate diagnoses and requires interpersonal skills like building rapport, providing reassurance and displaying empathy. A notable exception which incorporates this assessment is the Articulate Medical Intelligence Explorer (AMIE) system by Google,^18^ which fine-tunes an LLM for clinical reasoning, but is not available for use outside of Google Research. Studies that simulate patient agents, which can be used for practising communication skills, are less common. Assessment criteria for these largely focus on fidelity to assigned role and the ability to recall information.^19^,^23^ Few have incorporated analyses of dialogue realism and authenticity.^15 24^ Furthermore, qualitative evaluations tend to rely solely on medical expertise and fail to take patient perspectives into account. A more comprehensive investigation into the conversational abilities of LLM-based agents is needed before they can be deployed for medical training.

To address this investigative shortfall and better establish the opportunities and challenges of adopting modern LLMs for simulation-based teaching, we conducted a study to evaluate the fidelity of clinical interactions simulated using ChatGPT-based agents. ChatGPT is a popular language model by OpenAI that has been trained to follow user instructions. We selected an off-the-shelf model since it is easily accessible through web and programming interfaces. Moreover, fine-tuning billion-parameter models requires significant computational resources, which are unlikely to be available in academic settings. Our aim was to determine the realism with which the model can play the role of patients, doctors and examiners.

## Methods

We selected four paediatric scenarios typical of the undergraduate OSCE and prompted ChatGPT to simulate a patient agent, a doctor (student) agent and an examiner agent for each scenario. These agents are subsequently referred to as PatientGPT, DoctorGPT and ExaminerGPT. We simulated all three participants to fully explore the breadth of educational applications that could be supported with AI, such as peer-based learning (DoctorGPT) and providing narrative feedback (ExaminerGPT). Using prompts designed from the OSCE vignettes, PatientGPT and DoctorGPT were instructed to converse with each other. The simulated dialogues were then passed to ExaminerGPT for grading DoctorGPT’s performance. Finally, clinicians and patients familiar with the OSCE assessed the realism of the generated conversations and their utility as a teaching supplement. Clinicians also graded DoctorGPT’s performance, and their marking was compared with that of ExaminerGPT to measure the overlap between human and AI judgement.

We focused on AI–AI interactions instead of human–AI interactions, which can be difficult to standardise due to variability in human behaviour. We used the GPT-3.5 Turbo model in a zero-shot setting (i.e, without providing any examples of expected outputs) for all experiments. The model was accessed through the OpenAI application programming interface in Python. The scenarios were sourced from mock paediatrics OSCE stations for Year 4 MBChB students within the School of Medicine at the University of Leeds. They were chosen purposefully to reflect the communication objectives commonly assessed in OSCEs, including information giving, history taking and managing difficult or sensitive situations. The four stations are:

1. Exploring a mother’s concerns about the MMR vaccine’s potential link to autism, and its risks and benefits.
2. Explaining test results, and meningitis diagnosis and treatment to a baby’s father.
3. Taking paediatric history and explaining management for a benign heart murmur to a child’s father.
4. Taking an adolescent history from a teenage girl using the HEADSSS approach^25^ and explaining self-harm management.

For each scenario, we set up one simulation each of PatientGPT, DoctorGPT and ExaminerGPT. Station-specific instructions from the OSCE vignettes were converted to the second person to set agent roles. Additional instructions to elicit desirable behaviours were appended to the station instructions. These were identified by iteratively refining the role-setting prompts for each agent. PatientGPT was prompted to use colloquial language and filler words, express emotions and relate to life experiences, to enhance the realism of its responses. DoctorGPT was instructed to be sensitive and empathetic, provide comprehensive answers, summarise key points of the conversation and recommend relevant sources of information. No instructions about OSCE time limits were added to the prompts since the model did not have access to any external tools to keep time. The conversations were synthesised by prompting DoctorGPT to initiate a dialogue with PatientGPT. Responses were exchanged between the agents until a response signalling the end of the conversation was produced. The simulated interaction was then provided to ExaminerGPT to grade DoctorGPT’s performance. The grading included station criteria, communication skills, organisation skills and overall grade, all of which were taken from the OSCE information sheets. When no marking style was specified, we found that ExaminerGPT graded very leniently, so we added an additional instruction for strict grading.

Expert evaluation was done by three paediatric consultants and three expert patient volunteers involved in delivering the OSCE. The number of assessors was chosen to be consistent with similar recent studies.^15 16 24^ The protocol for evaluation was reviewed by the School of Medicine Research Ethics Committee, University of Leeds on 27 January 2024 (Reference: MREC 23-023). Informed consent was sought from all participants, and all identifiable data were anonymised before analysis. No demographic data were collected. Since the OSCE time limit was not considered during experimentation, the evaluators were asked to disregard conversation length while assessing the outputs. PatientGPT was judged on faithfulness to role, the ability to express emotions and perceived authenticity, using a 5-point scale (strong disagreement to strong agreement). DoctorGPT was judged on the ability to show empathy (same 5-point scale). Assessment for DoctorGPT also included communication and organisation skills, rated on a 4-point scale (poor to excellent). Evaluators also provided free-text feedback and rated the pedagogical utility of the simulated dialogues (same 5-point scale). Clinicians graded DoctorGPT’s performance using the same criteria and rubric as ExaminerGPT. Assessments were collected electronically using online forms. Weighted Cohen’s kappa^26^ was used to measure the level of agreement between each pair of clinicians, and between each clinician and ExaminerGPT. The prompts for agents, the simulated conversations and the free-text feedback are provided in the online supplemental material.

## Results

### PatientGPT

#### Qualitative attributes

**Figure 1:**
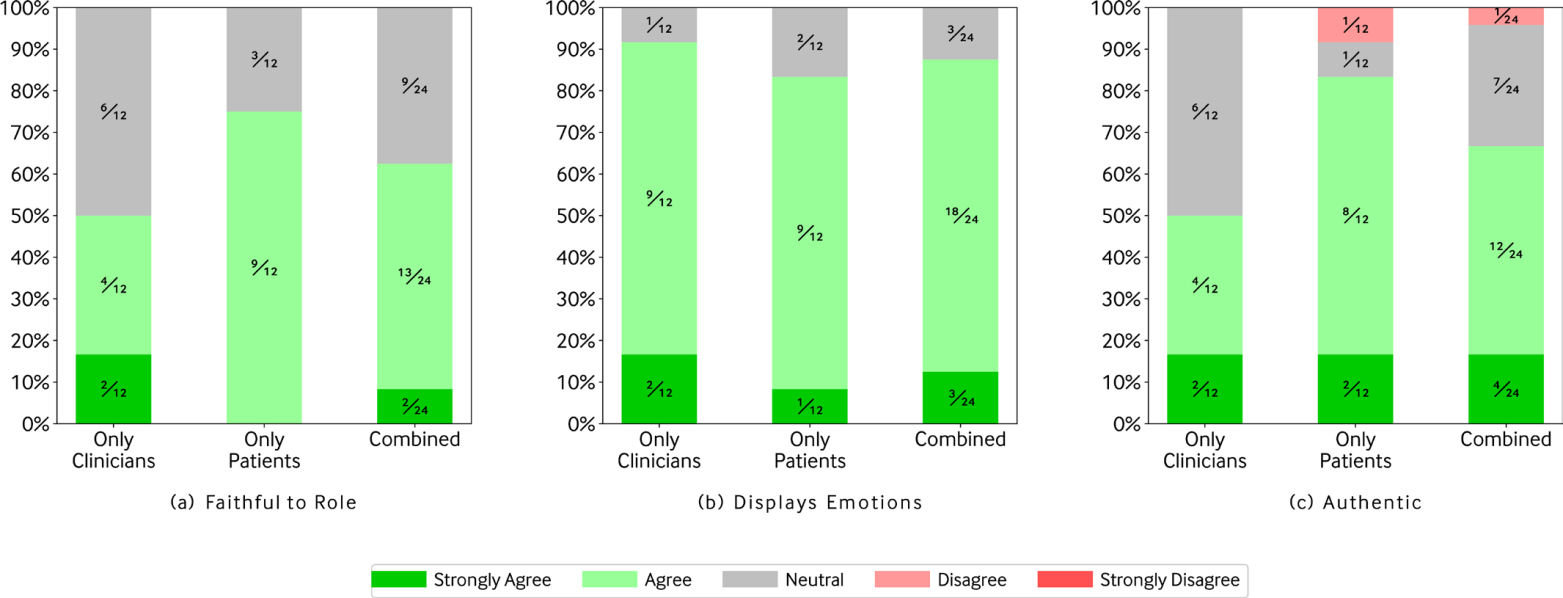
Expert evaluations for the PatientGPT agent across all scenarios. The 'Only Clinicians’ and 'Only Patients’ bars represent a sample size of 12, while the bar for 'Combined’ depicts percentages based on 24 samples.

Results for PatientGPT are presented in Figure 1. Each assessor group has a sample size of 12 (3 assessors × 4 stations), while the combined results have a sample size of 24 ((3+3) assessors × 4 stations). PatientGPT was judged to be moderately faithful to its assigned character (15/24 in agreement overall), with the patient assessment being more favourable than the clinician assessment (9/12 vs 6/12 in agreement, respectively). Similar results were obtained for the authenticity of the patient agent (16/24 in agreement overall), with higher approval from the patients than the clinicians (10/12 vs 6/12 in agreement, respectively). Judgement on the emotions expressed in PatientGPT’s responses was more consistent, with both groups reporting high agreement (11/12 and 10/12, respectively).

#### Free-text feedback

Some repetition and unnaturalness were reported in the free-text feedback for PatientGPT. An example of this echoing (from the benign heart murmur case) is shown below:

**DoctorGPT:** …That’s good to hear that Jack has been doing well and hasn’t had any significant symptoms since the viral infection.

**PatientGPT:** Yeah, it’s definitely a relief that Jack has been doing well and hasn’t had any more symptoms. I was a bit worried when the GP mentioned the murmur, you know?

It was also noted that responses by PatientGPT were over-eager and did not accurately mirror the hesitation shown by actual patients. This was especially relevant in the self-harm management scenario (sensitive content below):

**DoctorGPT:**

…Can you tell me a bit more about what happened and how you’re feeling now?

**PatientGPT:**

Yeah, sure. So, earlier tonight, I got into this big argument with my boyfriend. It was, like, really intense and we ended up breaking up. I was so upset and I just couldn’t see a life without him, you know? So, I did something stupid and cut my wrists with a razor blade. But now, I regret it and I don’t want to die anymore. I just want to forget this whole thing happened.

Clinicians additionally commented that unlike actual patients, PatientGPT was persuaded more easily to change its stance and opinions. This behaviour may be attributed to the issue of *sycophancy* in LLMs,^27^ where models trained on human feedback tailor responses by repeating or reinforcing user preferences (the user in this scenario being DoctorGPT). Other issues included occasional repetition of DoctorGPT’s jargon and overuse of colloquial expressions which diminished the perceived concern. However, both groups reported that PatientGPT’s responses were appropriate most of the time.

### DoctorGPT

#### Qualitative attributes

**Figure 2:**
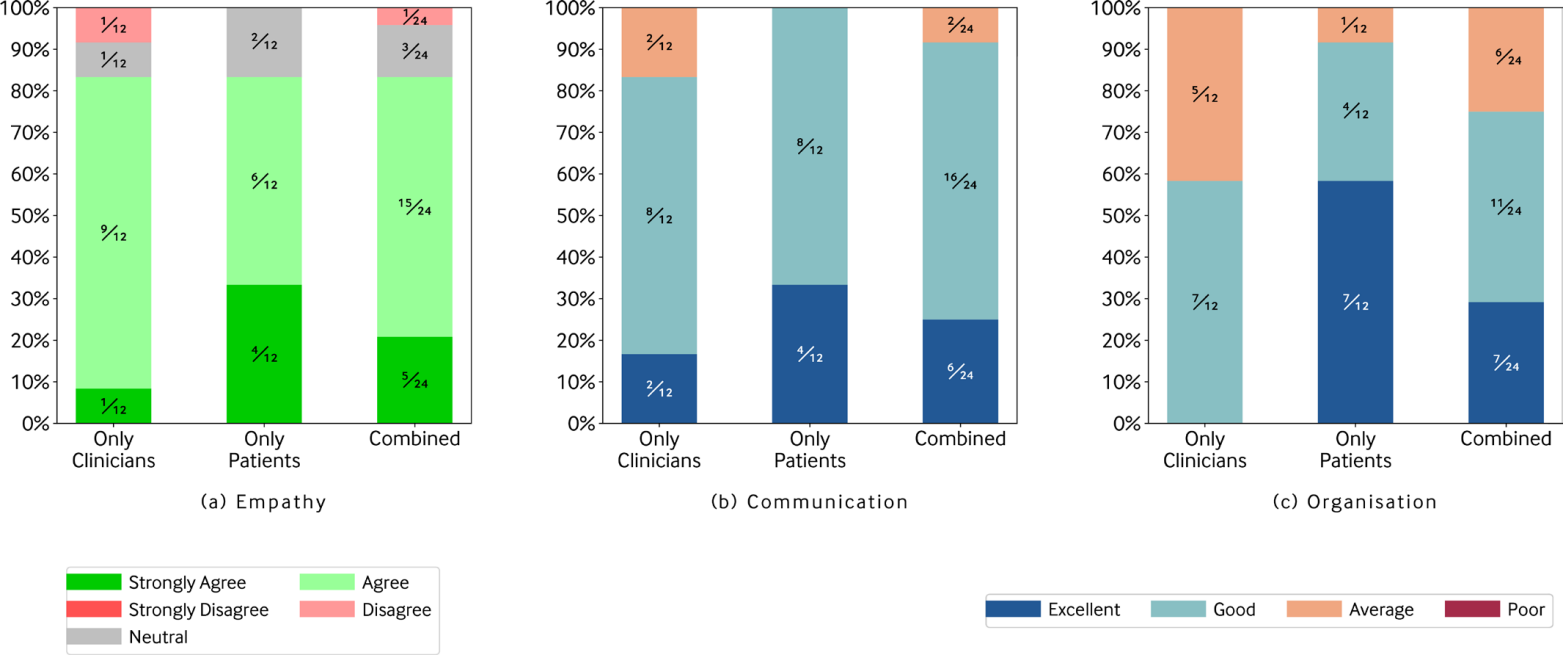
Evaluation for DoctorGPT’s empathy, communication skills and organisational skills. The 'Only Clinicians’ and 'Only Patients’ bars represent a sample size of 12, while the bar for 'Combined’ shows percentages corresponding to 24 samples.

Figure 2 reports the ratings received by DoctorGPT. The ‘patient only’ and ‘clinician only’ results have a sample size of 12 (3 assessors × 4 stations), while the combined results have a sample size of 24 ((3+3) assessors × 4 stations). DoctorGPT was reported to be an empathetic communicator overall (20/24 in agreement), with the patient ratings being slightly more varied across the 5-point scale than those from the clinicians (10/12 in agreement for both groups). Both groups favoured the second highest rating for the agent’s communication skills, with the patients tending to award slightly higher scores than the clinicians. Perceptions of the organisational approach of DoctorGPT were less consistent. Patients leaned towards the highest rating, while ratings from the clinicians were almost equally divided between ‘Average’ and ‘Good’.

#### Station grades

Clinicians also assessed station-specific criteria and provided an overall grade for DoctorGPT (Table 1) using a different 5-point scale (Clear fail, Borderline, Clear pass, Very good pass, Excellent pass). The rubric corresponding to the grades is provided in the online supplemental material. DoctorGPT received passing grades from all assessors in the MMR vaccine and heart murmur stations. Assessment for the meningitis diagnosis scenario was also in favour of (borderline) passing grades. There was no overlap in clinician marking for the self-harm scenario, but the (human) majority was again in favour of (different) passing grades.

**Table 1:** The overall grades awarded to DoctorGPT for each OSCE station

Station	Clinician 1	Clinician 2	Clinician 3	ExaminerGPT
MMR vaccine	Very good pass	Very good pass	Clear pass	Clear pass
Meningitis diagnosis	Borderline	Borderline	Clear fail	Borderline
Self-harm management	Borderline	Clear pass	Clear fail	Clear fail
Heart murmur	Clear pass	Clear pass	Clear pass	Borderline

OSCE, objective structured clinical examination.

#### Free-text feedback

Recurring themes in DoctorGPT’s feedback included wordy, repetitive answers which undermined realism and lack of summarisation of key points of the conversation. The agent also failed to recommend resources for further information, where needed. Some responses were reported to be formulaic and lacking personalisation. Patients additionally reported that DoctorGPT sometimes followed a tick-box approach, focussing on follow-up questions rather than letting PatientGPT elaborate concerns, which was deemed somewhat unnatural. However, DoctorGPT was judged by both assessor groups to be good at providing reassurance and explanations, despite occasionally responding with unnecessary jargon. This was observed in the MMR vaccine scenario, where the agent included journal names and exact statistics when debunking the link between the vaccine and autism:

**DoctorGPT:**

…One notable study published in the Journal of the American Medical Association (JAMA) in 2002 included over 500,000 children and found no increased risk of autism following the MMR vaccine. Another study published in the New England Journal of Medicine in 2019 analyzed data from over 650,000 children and also found no association between the MMR vaccine and autism…

Additionally, while DoctorGPT received a (borderline) passing grade for the self-harm management case, it failed to mention drug use or sexual relationships, which are vital aspects of the HEADSSS history. This is an artefact of continuing efforts to detoxify LLMs by restricting topics of conversation.^28^

### ExaminerGPT

**Figure 3:**
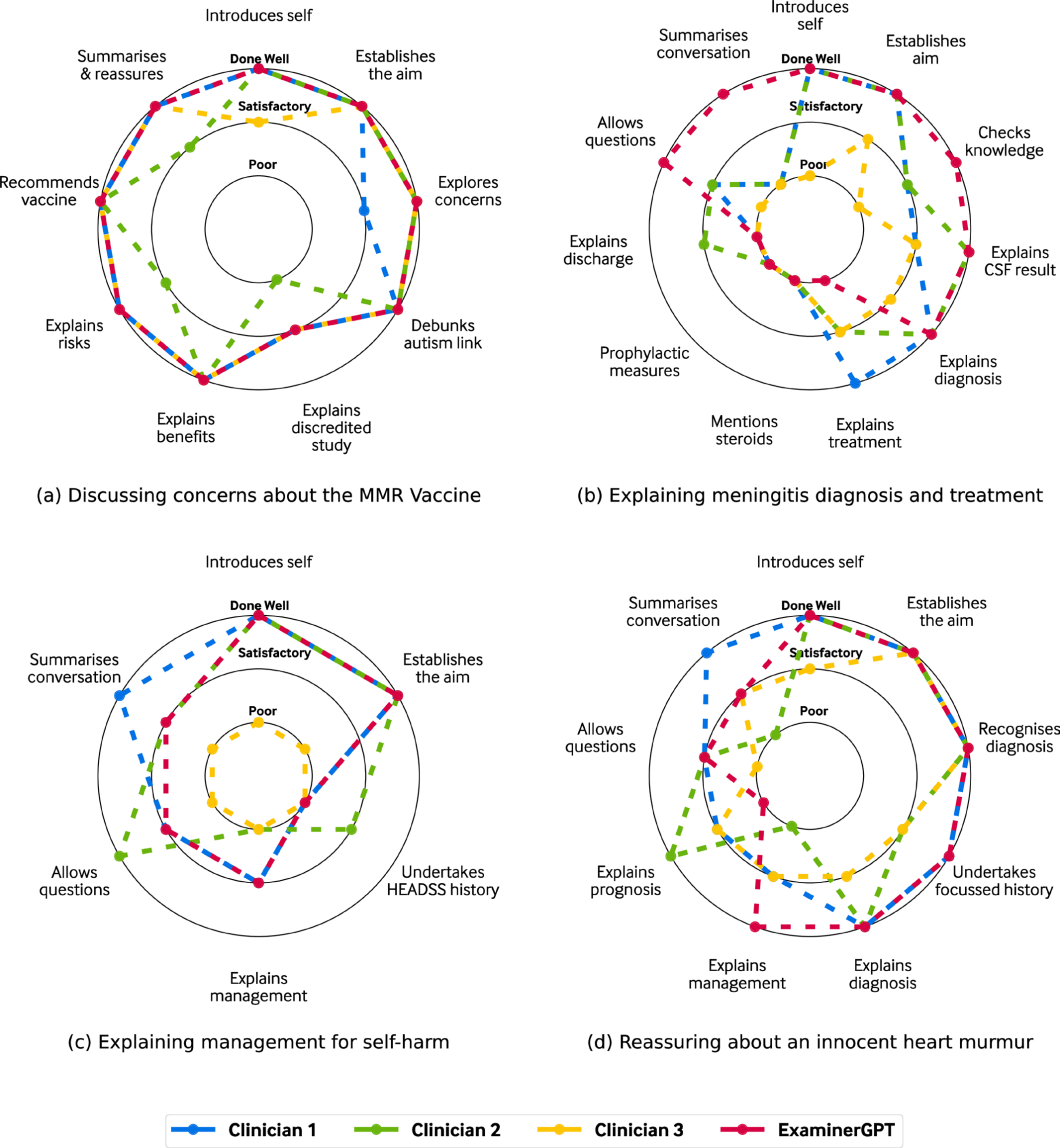
Station-specific marking for DoctorGPT done by three human clinicians and ExaminerGPT. The agent had good overlap with clinician judgement most of the time, but struggled in cases where a stricter assessment was requisite.

ExaminerGPT was assessed by checking the overlap between its markings and human judgement. Both clinicians and ExaminerGPT marked DoctorGPT on station-specific criteria using a 3-point scale (Done well, Satisfactory, Not attempted or Poor). Figure 3 shows how the evaluators marked DoctorGPT for each station. Across the four scenarios, the average agreement between ExaminerGPT–Clinician pairs (κ=0.54*,*0.36*,*0.26; mean=0.39) was higher than the average agreement between Clinician–Clinician pairs (κ=0.42*,*0.22*,*0.35; mean=0.33). For overall grade (Table 1), ExaminerGPT’s judgement had reasonable overlap with the clinicians for binary pass/fail decisions, but overlap with the majority judgement occurred in only one case (meningitis diagnosis). The justifications produced by ExaminerGPT for its scoring are available in the online supplemental material.

### Pedagogical utility

**Figure 4:**
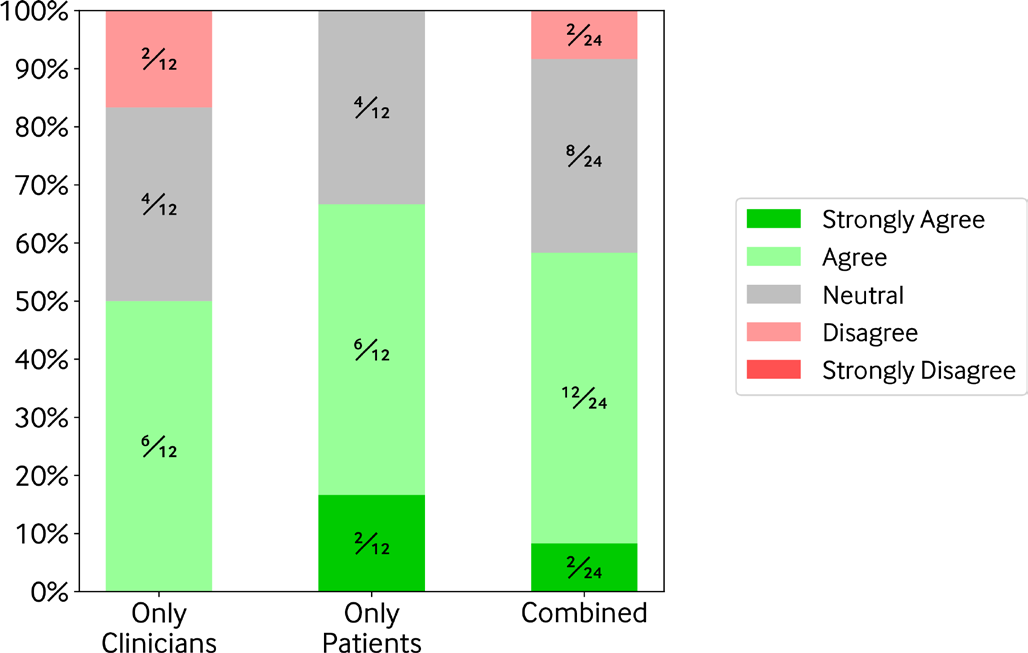
Assessor responses on the usefulness of the simulated dialogues as a learning aid. The 'Only Clinicians’ and 'Only Patients’ bars represent a sample size of 12, while the bar for 'Combined’ shows percentages corresponding to 24 samples.

The final question posed to assessors addressed the potential utility of the simulated interactions in medical training. Figure 4 presents assessor agreement with the statement: ‘*This conversation can be used for educational purposes*’. Just over half of the combined assessors thought the conversations could be a useful learning resource (14/24 in agreement), with the patient response being more favourable than the clinician response (8/12 vs 6/12 in agreement).

## Discussion

Overall, we found reasonable evidence of realism offered by the ChatGPT-based agents. PatientGPT showed good ability to express emotions while responding. The agent was faithful to its character for most of the interaction and moderately representative of true patients. DoctorGPT was good at expressing empathy and offering explanations, but failed to address sensitive aspects of clinical communication. The agent also had scope for improvement in its organisational skills. However, DoctorGPT attained passing grades in all scenarios (based on majority clinician ratings). ExaminerGPT displayed high agreement with the human clinicians, but tended to mark leniently. There was moderate support for using such simulated conversations in clinical teaching.

Our findings largely corroborate recent studies that showcase the potential of LLMs in supporting simulation-based training.^19 24^ However, many works restrict assessment of agents to rigid measures like diagnostic correctness^15^,^17^ or the ability to recall role-specific information.^20 22 23^ In contrast, we have sought qualitative assessments as well as free-text feedback, which provide greater insight into the behaviour of the GPT agents. A notable issue which undermined the perceived realism of both agents was a tendency to frequently agree with and repeat each other’s statements, resulting in some unnatural echoing and verbosity. This behaviour is likely amplified due to the AI–AI interaction setup and would be less pronounced in the human–AI interactions. Both assessor groups reported that PatientGPT was more amenable to changing its opinion than real patients. Although this *sycophancy* can limit the applicability of GPT-based agents for simulating diverse scenarios with angry or difficult patients, we found limited discussion on this issue in the existing literature.^24^ Learning to personalise communication based on patient characteristics is also essential for experiential learning.^29^ Patients reported that DoctorGPT tended to favour formulaic approaches to conversation. Despite being a potential barrier to building trust and rapport, few studies have considered this facet in their assessment.^18^ Finally, while existing literature relies solely on medical expertise to evaluate LLM-based agents, the current study also incorporates expert patient perspectives. Notable disparities between assessments done by the two assessor groups were observed for PatientGPT’s faithfulness to role and authenticity, and for DoctorGPT’s organisational skills. This was despite both groups being familiar with the OSCE and highlights the subjectivity inherent in discourse evaluation, as well as the importance of including diverse perspectives.

While the role-playing capabilities of ChatGPT are limited for complex scenarios, it can still serve as a valuable learning tool, particularly for formative assessment and feedback. Reviewing representative conversational transcripts can help learners improve their interviewing skills.^30^ These dialogues can also be used to fine-tune smaller language models for role-playing, which can then be deployed in resource-constrained settings. Conversely, scenarios scored poorly by assessors could be used to help learners identify and reflect on missed opportunities. LLMs offer a scalable method to educate students about consultation skills in the clinical workplace, encompassing both common and rare scenarios. However, there are intrinsic limits to the consistency and reliability of their outputs,^31^ which necessitate a continuing need for human oversight of models deployed in pedagogical settings. Our study highlights the growing need to train faculty in AI-enhanced teaching. Finally, we note some limitations in our work. Our findings are likely to have limited generalisability in diverse cultural settings due to the small number of scenarios simulated, and a relatively small assessor group. They are also limited to the written format and do not incorporate non-verbal aspects of communication such as tone of voice or body language. However, our work helps establish the utility of AI-generated dialogues for medical training, and could guide future work that integrates such text with speech or avatars. Furthermore, we have not considered the OSCE time limit in our experiments, and results will likely differ when this aspect is incorporated. Future studies may explore incorporation of real-world constraints and novel prompting strategies to reduce sycophancy and repetition in GPT-generated dialogues.

## Supplementary material

10.1136/archdischild-2025-329846 online supplemental file 1

## Data Availability

All data relevant to the study are included in the article or uploaded as supplementary information.
